# Saikosaponin A inhibits IL-1β-induced inflammatory mediators in human osteoarthritis chondrocytes by activating LXRα

**DOI:** 10.18632/oncotarget.21495

**Published:** 2017-09-30

**Authors:** Hang Gao, Yanyan Song, Dongsong Li, Wei Feng, Jianguo Liu

**Affiliations:** ^1^ Department of Bone and Joint Surgery, The First Hospital of Jilin University, Changchun, Jilin Province, China; ^2^ Department of Nephrology, The Second Hospital of Jilin University, Changchun, Jilin Province, China

**Keywords:** osteoarthritis chondrocyte, IL-1β, NF-κB, LXRα

## Abstract

Saikosaponin a (SSa), one of the main active components of *Bupleurum falcatum*, has been reported to have anti-inflammatory effect. In the present study, we investigated the anti-inflammatory effect of SSa on IL-1β-stimulated human osteoarthritis chondrocytes. The cells were pretreated with SSa 12 h before IL-1β treatment. The production of PGE2 and NO were detected by ELISA and Griess method. The levels of MMP1, MMP3, and MMP13 were measured by ELISA and qRT-PCR. The expression of NF-κB and LXRα were tested by western blot analysis. The results showed that SSa inhibited IL-1β-induced PGE2 and NO production in a concentration-dependent manner. SSa also suppressed IL-1β-induced MMP1, MMP3, and MMP13 production. Furthermore, SSa significantly attenuated IL-1β-induced phosphorylation levels of NF-κB p65 and IκBα. SSa also up-regulated the expression of LXRα. The inhibition of SSa on PGE2, NO, MMP1, MMP3, and MMP13 production were reversed by LXRα siRNA or GGPP, the inhibitor of LXRα. In conclusion, our results demonstrated that SSa inhibited inflammatory responses in human chondrocytes *in vitro*. SSa might be a potential therapeutic drug for osteoarthritis.

## INTRODUCTION

Osteoarthritis (OA), a common form of arthritis, is characterized by degradation and destruction of cartilage matrix and inflammatory responses in chondrocytes [1]. The pathophysiology of OA is complex. However, accumulating evidence suggested that inflammation played a critical role in the development of OA [2, 3]. IL-1β is an important inflammatory cytokine that involved in the pathologic process of OA [4]. IL-1β could induce the activation of NF-κB signaling pathway, which leads to the release of inflammatory mediators [5]. These inflammatory mediators, such as PGE2, NO, and MMP, lead to articular cartilage damage [6]. In recent years, non-steroidal anti-inflammatory drugs were often used in the treatment of OA [7]. However, due to the numerous side effects, the development of effective and safe drugs to treat OA is urgently needed.

Saikosaponin a (SSa), a bioactive ingredient isolated from *Bupleurum falcatum*, has been known to exhibit anti-inflammatory effect [8]. Previous study showed that SSa could inhibit LPS-induced inflammatory cytokines production in RAW264.7 cells [9]. SSa also inhibited carrageenan-induced paw edema in rats and acetic acid-induced vascular permeability in mice [8]. Furthermore, SSa has been reported to inhibit oxidative stress and inflammation in LPS-stimulated human umbilical vein endothelial cells [10]. SSa has been reported to inhibit inflammation in hypertrophied 3T3-L1 adipocytes [11]. However, the effects of SSa on IL-1β-stimulated chondrocytes has not been reported. In the present study, we aimed to investigate the anti-inflammatory effect of SSa on IL-1β-stimulated human osteoarthritis chondrocytes and clarify the underling mechanism.

## RESULTS

### SSa did not affect the viability of human osteoarthritis chondrocytes

**Figure 1:**
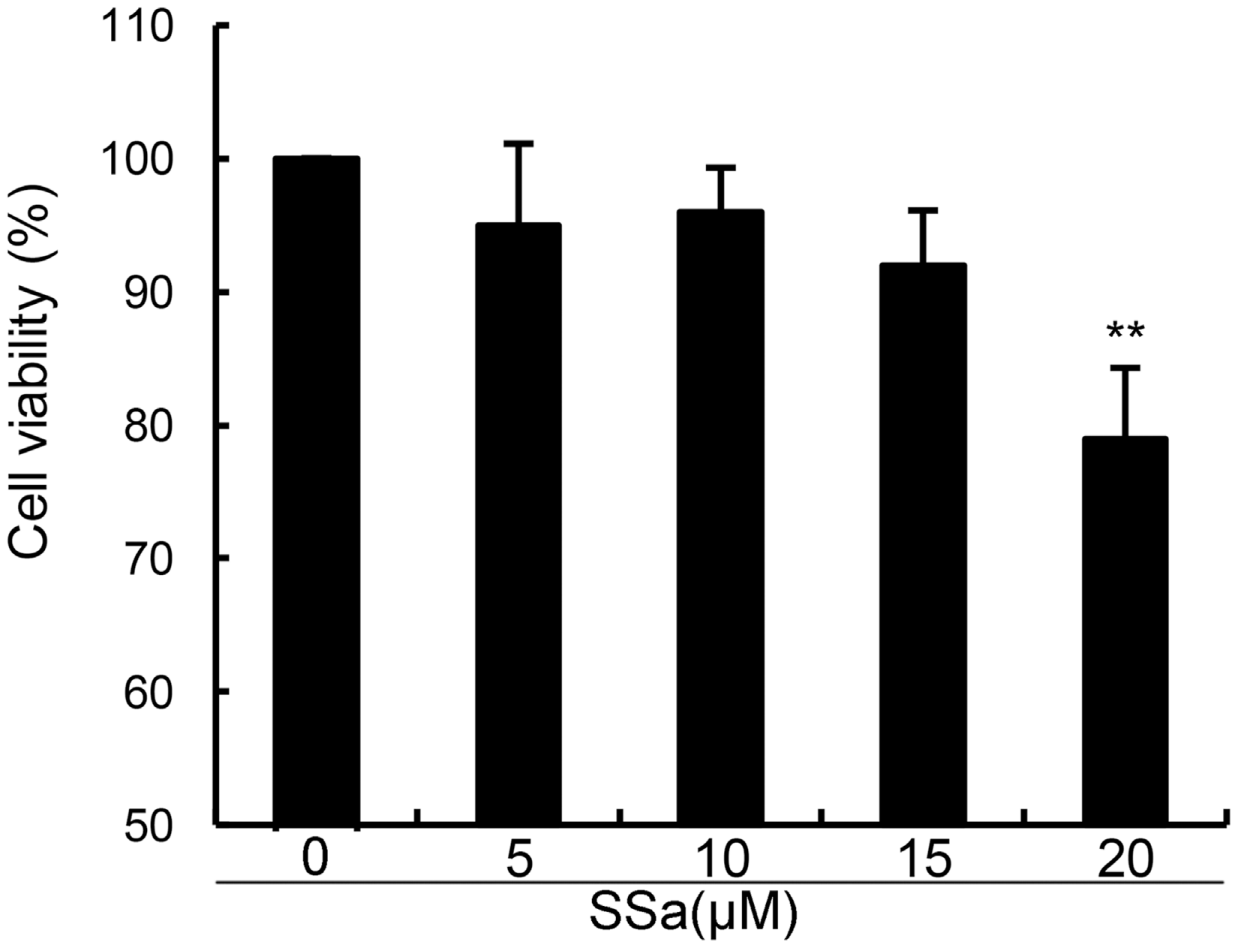
Effects of SSa on the cell viability of chondrocytes The values presented are the means ± S.E.M. of three independent experiments. ^*^P < 0.05, **P < 0.01 *vs.* control group.

In this study, we firstly detected the effects of SSa on the viability of human osteoarthritis chondrocytes. As shown in Figure 1, compared with the control group, SSa at concentration of 5, 10, 15μM did not affect the viability of human osteoarthritis chondrocytes (Figure 1). Therefore, the concentration of 5, 10, 15μM were used in the subsequent study.

### SSa inhibits IL-1β-induced NO and PGE2 production in human osteoarthritis chondrocytes

**Figure 2:**
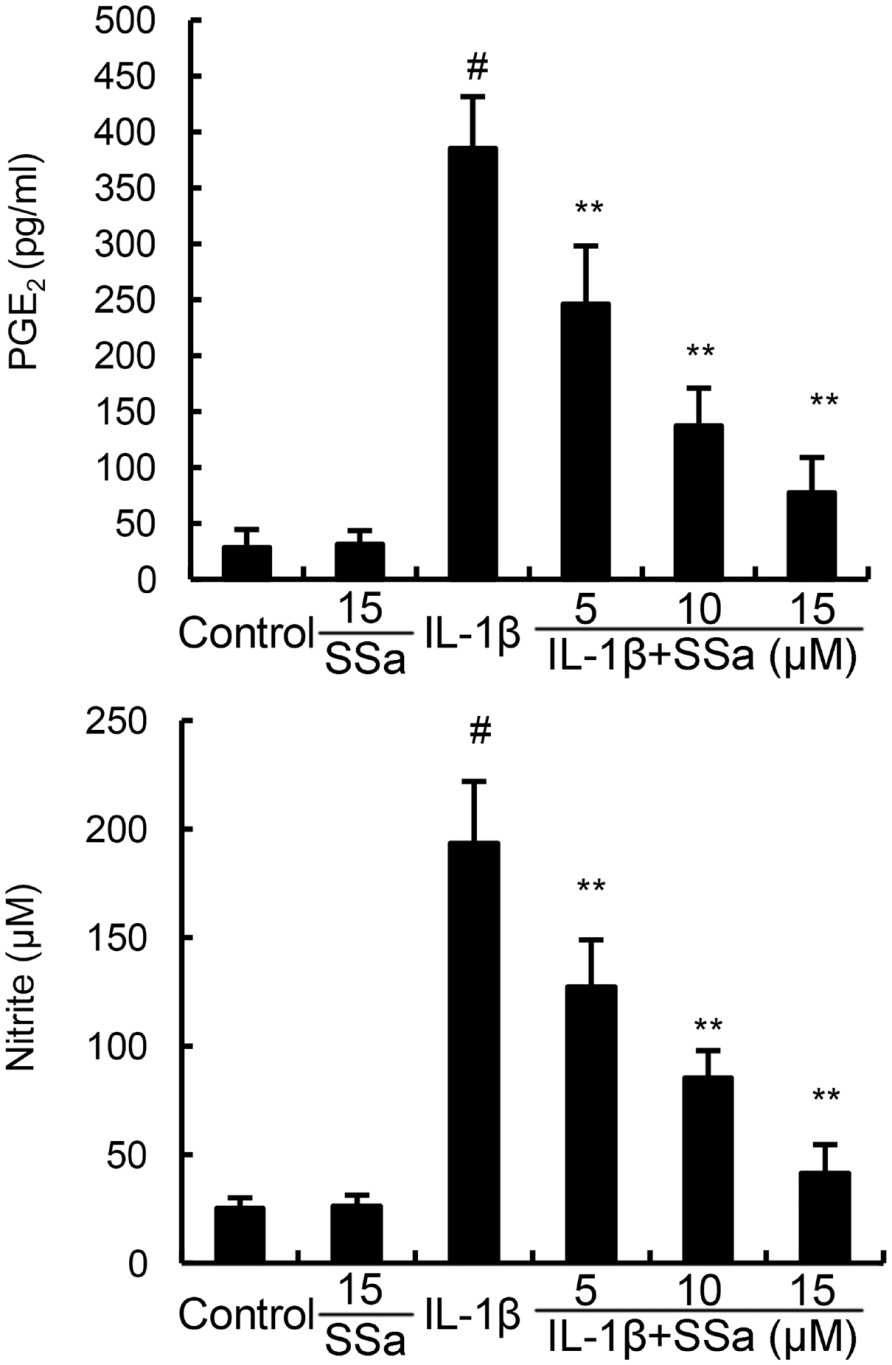
SSa inhibits IL-1β-induced NO and PGE_2_ production The data presented are the means ± S.E.M. of three independent experiments. ^#^*P* < 0.05 *vs.* control group; *P < 0.05, **P < 0.01 *vs.* IL-1β group.

We investigated the effects of SSa on IL-1β-induced NO and PGE2 production to test whether SSa exhibited anti-inflammatory effects in human osteoarthritis chondrocytes. As shown in Figure 2, SSa alone did affect the production of NO and PGE2. Compared with the control group, the levels of NO and PGE2 increased significantly in IL-1β-stimulated human osteoarthritis chondrocytes. However, IL-1β-induced NO and PGE2 production were significantly inhibited by treatment of SSa.

### Effects of SSa on IL-1β-induced MMP1, MMP3, and MMP13 expression

**Figure 3:**
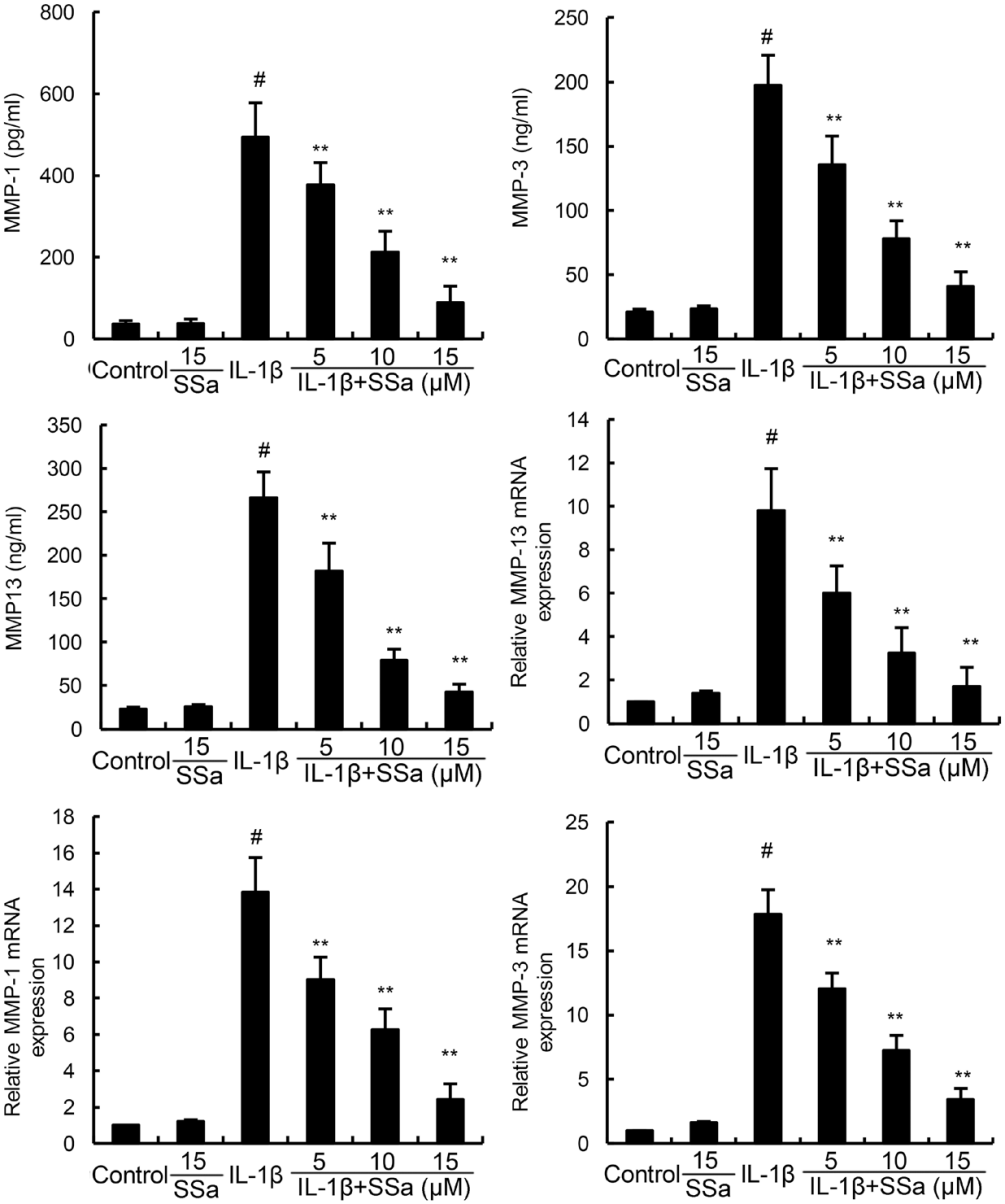
SSa inhibits IL-1β-induced MMP1, MMP3, and MMP13 production The data presented are the means ± S.E.M. of three independent experiments. ^#^*P* < 0.05 *vs.* control group; *P < 0.05, **P < 0.01 *vs.* IL-1β group.

We investigated the effects of SSa on IL-1β-induced MMP1, MMP3, and MMP13 production by ELISA and qRT-PCR. As shown in Figure 3, SSa alone did affect the expression of MMP1, MMP3, and MMP13. Compared with the control group, the mRNA and protein levels of MMP1, MMP3, and MMP13 increased significantly in IL-1β-stimulated human osteoarthritis chondrocytes. However, IL-1β-induced MMP1, MMP3, and MMP13 production were significantly inhibited by treatment of SSa (Figure 3).

### Effects of SSa on IL-1β-induced NF-κB activation in human osteoarthritis chondrocytes

**Figure 4:**
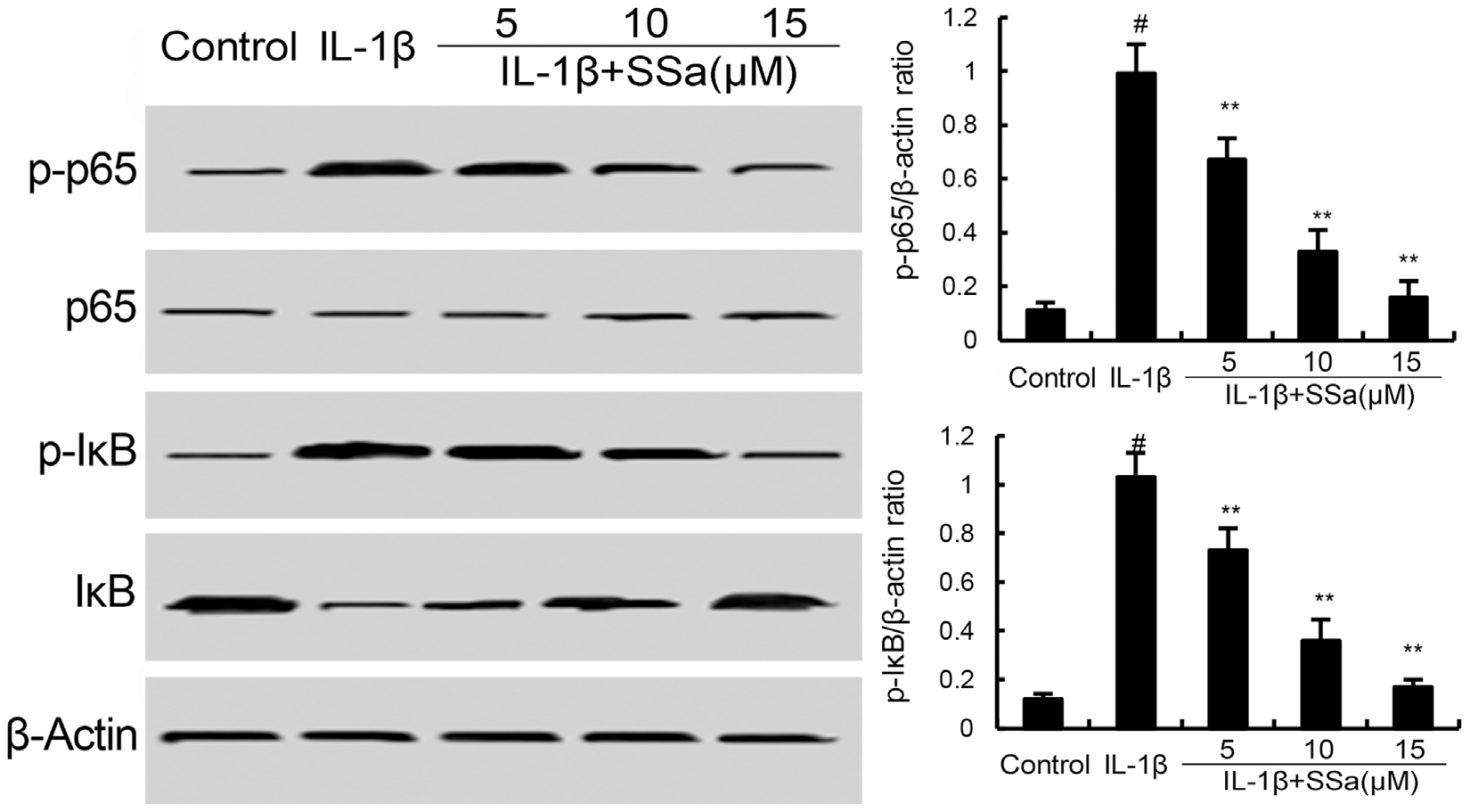
SSa inhibits IL-1β-induced NF-κB activation and IκBα degradation The values presented are the means ± S.E.M. of three independent experiments. ^#^*P* < 0.05 *vs.* control group; *P < 0.05, **P < 0.01 *vs.* IL-1β group.

We investigated the effects of SSa on IL-1β-induced NF-κB activation to test the anti-inflammatory mechanism of SSa in human osteoarthritis chondrocytes. As shown in Figure 4, compared with the control group, the levels of phosphorylation of NF-κB p65 and IκBα increased significantly in IL-1β-stimulated human osteoarthritis chondrocytes. However, IL-1β-induced NF-κB activation were significantly inhibited by treatment of SSa.

### Effects of SSa on LXRα expression

**Figure 5:**
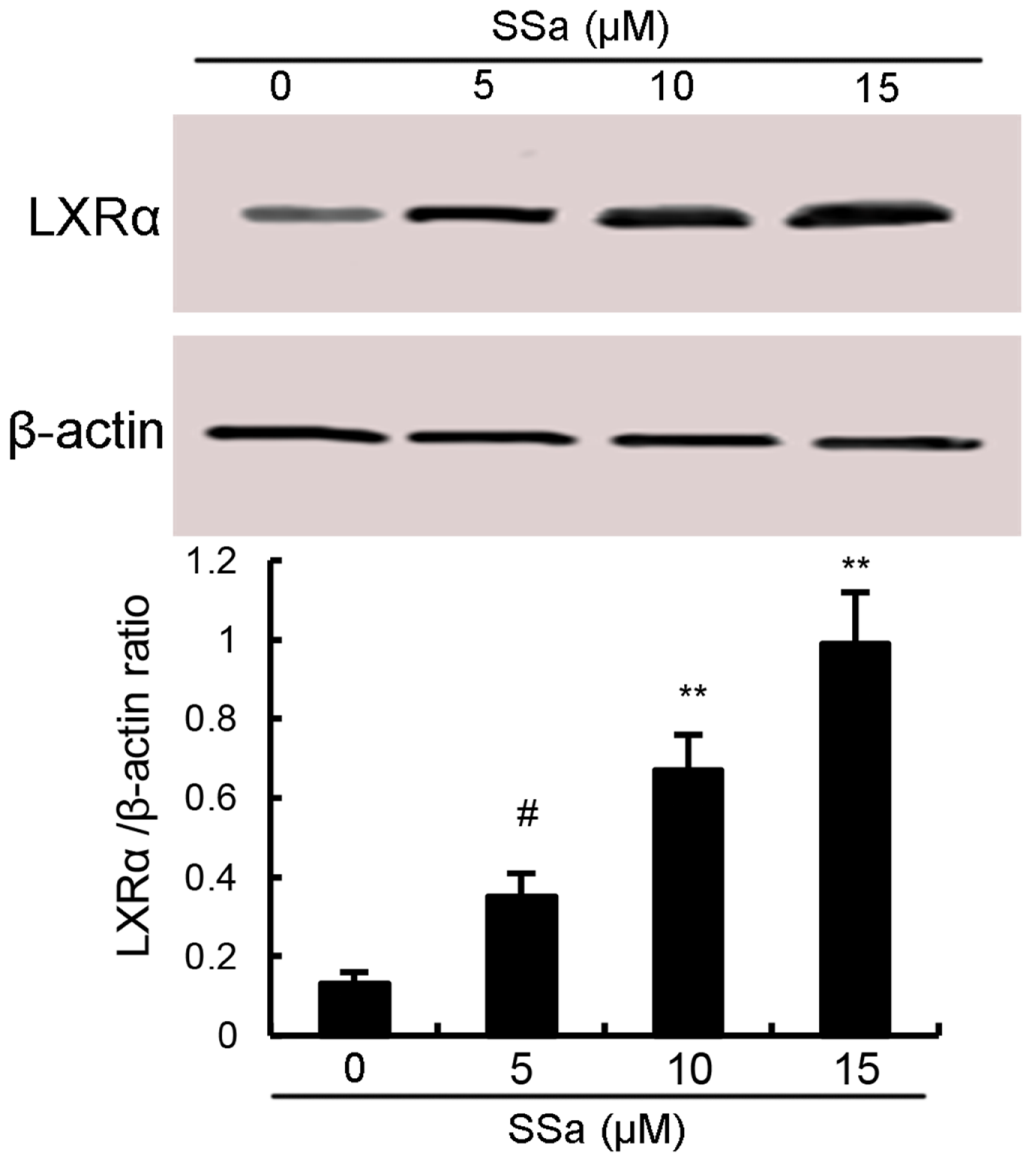
Effects of sesamin on LXRα expression The values presented are the means ± S.E.M. of three independent experiments. ^#^*P* < 0.05 *vs.* control group.

LXRα, the members of the nuclear hormone receptor superfamily, has been reported to have the ability to regulate the activation of NF-κB [12]. Therefore, in the present study, the effects of SSa on LXRα expression were detected by western blot analysis. As shown in Figure 5, SSa up-regulated the expression of LXRα in a dose-dependent manner.

### The anti-inflammatory effects of SSa is regulated by LXRα

**Figure 6:**
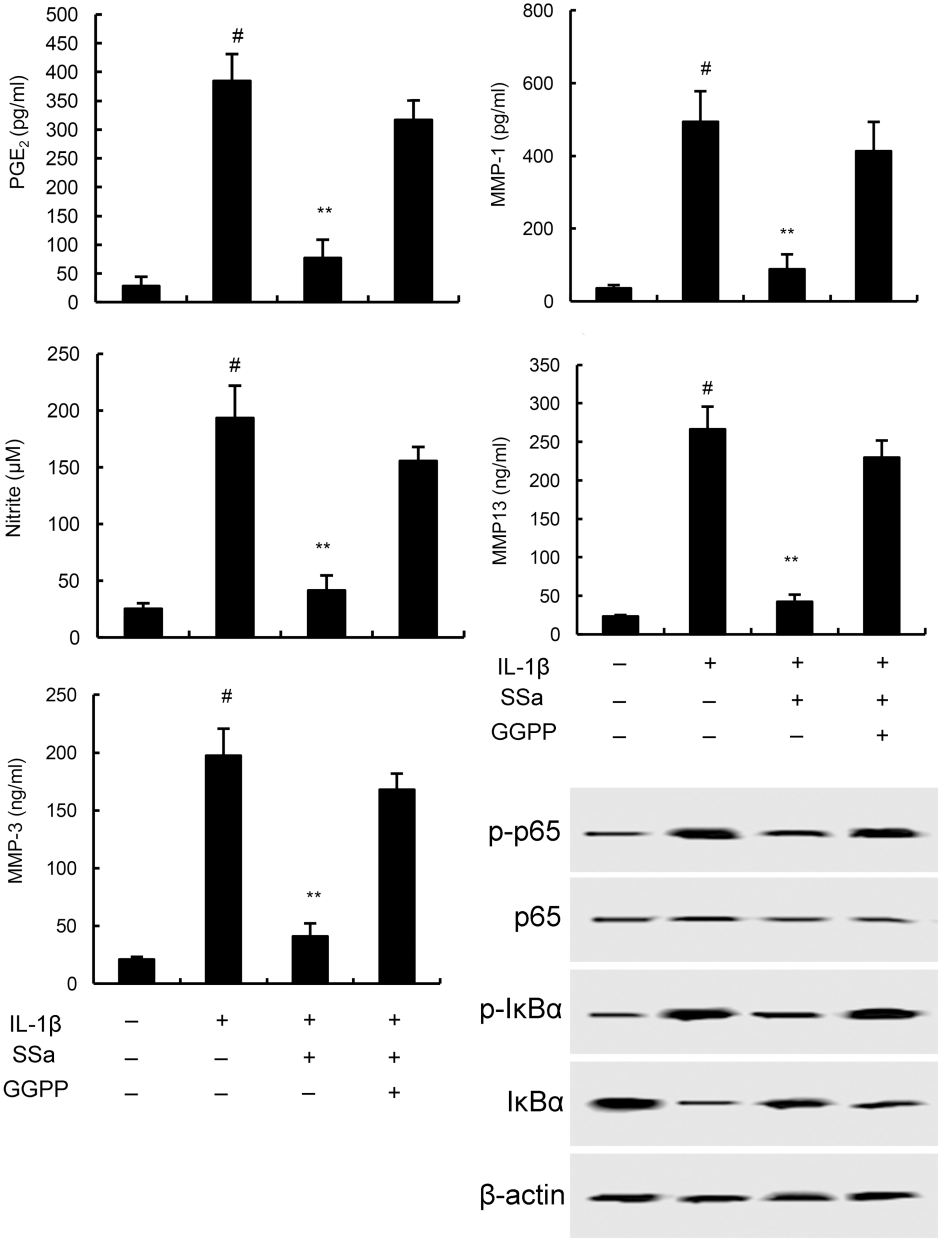
Effects of LXRα inhibitor GGPP (20 μM) on the anti-inflammatory effects of SSa The values presented are the means ± SEM of three independent experiments. #p < 0.05 vs. control group; *p < 0.05, **p < 0.01 vs. LPS group.

**Figure 7:**
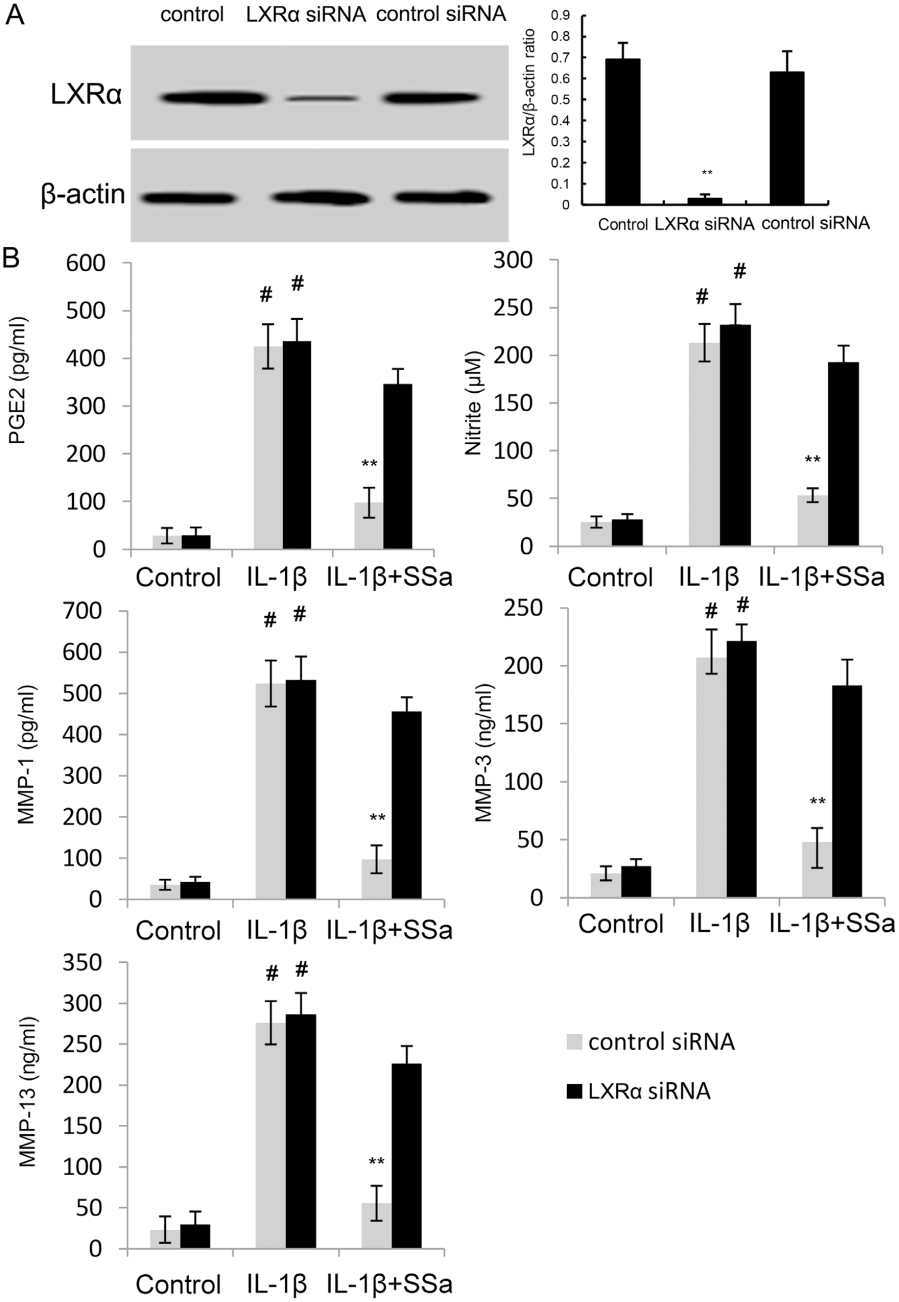
**(A)** Effects of siRNA on LXRα expression was detected by western blot analysis. **(B)** Effects of LXRα siRNA on the anti-inflammatory effects of SSa. The values presented are the means ± SEM of three independent experiments. #p < 0.05 vs. control group; *p < 0.05, **p < 0.01 vs. LPS group.

To further investigate the anti-inflammatory mechanism of SSa, LXRα was blocked by its inhibitor GGPP. As shown in Figure 6, the inhibition of SSa on NO, PGE2, MMP1, MMP3, and MMP13 production, as well as NF-κB activation were reversed by GGPP. The results indicated that SSa inhibited IL-1β-induced inflammation in human osteoarthritis chondrocytes by activating LXRα (Figure 6). Furthermore, the inhibition of SSa on NO, PGE2, MMP1, MMP3, and MMP13 production were reversed when LXRα was knockdown (Figure 7B).

## DISCUSSION

SSa has been reported to have anti-inflammatory effect [13]. Previous study showed that SSa could inhibit LPS-induced inflammatory response by inducing LXRα activation [14]. However, whether SSa could inhibit IL-1β-induced inflammatory response remain unclear. The aim of this study was to assess the anti-inflammatory effects of SSa on osteoarthritis. In the present study, we found that SSa significantly inhibited IL-1β-induced inflammatory response in chondrocytes. The anti-inflammatory mechanism of SSa was through activating LXRα, which subsequently inhibited IL-1β-induced NF-κB activation.

Many *in vitro* and *in vivo* studies demonstrated that inflammation play a fundamental role in the damage of articular tissues [15, 16]. The release of inflammatory mediators, such as PGE2 and NO result in destruction of the articular joint, leading to development of OA [6, 17]. Furthermore, these inflammatory mediators could induce the production of MMPs, which could induce degradation of ECM in OA articular cartilage [18]. Inhibition of these inflammatory mediators and MMPs could attenuate the development of OA [19, 20]. In the present study, we found that SSa significantly inhibited IL-1β-induced PGE2 and NO, as well as MMP1, MMP3, and MMP13 production. NF-κB is transcription factor that regulates a large body of inflammatory genes expression [10, 21, 22]. Previous study suggested that NF-κB could be used as a potential therapeutic target in OA [23]. Inhibition of NF-κB activation could attenuate pain of OA [24, 25]. In the present study, our results showed that SSa significantly inhibited IL-1β-induced NF-κB activation in chondrocytes. These results indicated that SSa inhibited IL-1β-induced inflammation in chondrocytes by inhibiting NF-κB activation.

LXRα is a nuclear receptor that induces genes controlling cholesterol homeostasis and lipogenesis [26]. It is a ligand-activated transcription factor that recently has been reported to have anti-inflammatory effects [27]. A large body of studies showed that many natural compounds could activate LXRα to exhibit its anti-inflammatory effects [14, 28]. Previous study showed that activating LXRα could attenuate pain in a rat osteoarthritis model [29]. In addition, study showed that LXRα agonist had the ability to inhibit NF-κB activation. To clarify the anti-inflammatory mechanism of SSa, we detected whether SSa could activate LXRα. Our results showed that SSa significantly up-regulated the expression of LXRα. Furthermore, our results showed that blocking LXRα, the anti-inflammatory effects of SSa were inhibited. Taken together, our results showed that SSa exhibited its anti-inflammatory effects by activating LXRα.

In conclusion, in the present study, we showed that SSa significantly inhibited IL-1β-induced inflammatory response by inhibiting NF-κB activation. Furthermore, SSa has been found to up-regulate the expression of LXRα, which subsequently inhibited the activation of NF-κB. SSa may be a potential therapeutic agent for osteoarthritis.

## MATERIALS AND METHODS

### Chemicals and reagents

SSa (purity>98%) was purchased from the National Institute for the Control of Pharmaceutical and Biological Products (Beijing, China). Geranyl geranyl pyrophosphate (GGPP) was purchased from Sigma Chem. (St. Louis, USA). Recombinant human IL-1β, MMP1, MMP3, MMP13, and PGE2 ELISA kits were purchased from R&D systems (Minneapolis, MN, USA). Antibodies for LXRα, IκBα, and NF-κB were purchased from Santa Cruz Biotechnology (Santa Cruz, CA, USA). NO detection kit was purchased from Nanjing Jiancheng Bioengineering Institute. (Nanjing, China).

### Cell culture and treatment

The experiments were done in accordance with the guidelines established by the Jilin University Animal Care and Use Committee. The protocols were reviewed and approved by the committee. OA human cartilage tissues were obtained from 12 OA patients. Primary human osteoarthritis chondrocytes were isolated as described previously [30]. Then, the cells were cultured in DMEM containing 10% fetal bovine serum. The cells were pretreated with SSa (5, 10, 15μM) 12 h before IL-1β treatment. For LXRα inhibitory experiment, the cells were pretreated with or without 20 μM GGPP for 2 h, then the cells were pretreated with SSa (5, 10, 15μM) 12 h before IL-1β treatment. Cells between passages 1 to 3 were used in this study.

### Cell viability

The effects of SSa on the viability of human osteoarthritis chondrocytes were detected by MTT assay. In brief, human osteoarthritis chondrocytes were cultured in 96-well plate (5×10^3^cells/well) for 12 h. Then, the cells were treated with different concentrations of SSa. 24 h later, the cells were incubated with MTT for 4 h and the insoluble formazan product was dissolved in DMSO. Finally, the optical density was read using a microplate reader.

### Inflammatory mediator assay

The level of NO in the culture medium was detected by Griess reagent (Nanjing Jiancheng Bioengineering Institute, Jiangsu, China) according to the manufacturer’s instructions. The levels of PGE2, MMP1, MMP3, and MMP13 in the culture medium were detected by ELISA kits (R&D systems, Minneapolis, MN, USA) according to the manufacturer’s instructions.

### Western blot analysis

The cells were pretreated with SSa (5, 10, 15μM) 12 h before IL-1β (10 ng/ml) treatment. 1 h later, the cells were incubated with 200 μl cell lysis buffer (Beyotime, China) for 5min and subjected to centrifugation with 12000 g/min for 10min. Protein concentration were detected by BCA method. Equal amount of proteins were separated on 12% SDS-PAGE and transferred to PVDF membranes. Then, the membranes were blocked with 3% BSA and incubated with primary antibodies and secondary antibodies. Finally, the membranes were visualized by ECL detection reagents and imaged with the Pxigel imaging system.

### RNA extraction and qRT-PCR

Total RNA was extracted using the RNA simple total RNA kit (Tiangen, China). The cDNA was synthesized using a primescript RT reagent kit (Takara, Japan). Primers were obtained from Sangon Biotech Co. Ltd (Shanghai, China). Synthesized cDNA used for RT-PCR was performed with a 7500 real-time PCR system (Applied Biosystems, Carlsbad, CA, USA) as following: 50 °C for 2 min and 95°C for 10min followed by 40 cycles of 95 °C for 15 s and 60 °C for 1 min.

### Cells transfection with siRNA

Chondrocytes were transfected with siRNA-LXRα (100 nM) or control siRNA (100 nM) with Lipofectamine 2000 transfection reagent (Thermo, USA) for 24 h according to the manufacturer’s instructions. Then, the cells were incubated with SSa and stimulated with IL-1β. The inhibition of siRNA on LXRα expression was detected by western blot analysis.

### Statistical analysis

All the values were presented as the mean ± S.E.M. The differences between groups were analyzed using one-way ANOVA followed by post hoc analyses using the Tukey test. P <0.05 were considered to indicate statistical significance.
